# Microbial Virulence as an Emergent Property: Consequences and Opportunities

**DOI:** 10.1371/journal.ppat.1002136

**Published:** 2011-07-21

**Authors:** Arturo Casadevall, Ferric C. Fang, Liise-anne Pirofski

**Affiliations:** 1 Department of Microbiology & Immunology and Medicine, Albert Einstein College of Medicine, Bronx, New York, United States of America; 2 Departments of Laboratory Medicine and Microbiology, University of Washington School of Medicine, Seattle, Washington, United States of America; The Fox Chase Cancer Center, United States of America

Although an existential threat from the microbial world might seem like science fiction, a catastrophic decline in amphibian populations with the extinction of dozens of species has been attributed to a chytrid fungus [1], [2], and North American bats are being decimated by *Geomyces destructans*, a new fungal pathogen [3]. Hence, individual microbes can cause the extinction of a species. In the foregoing instances, neither fungus had a known relationship with the threatened species; there was neither selection pressure for pathogen attenuation nor effective host defense. Humans are also constantly confronted by new microbial threats as witnessed by the appearance of HIV, SARS coronavirus, and the latest influenza pandemic. While some microbial threats seem to be frequently emerging or re-emerging, others seem to wane or attenuate with time, as exemplified by the decline of rheumatic heart disease [4], the evolution of syphilis from a fulminant to a chronic disease [5], and the disappearance of “English sweating sickness” [6]. A defining feature of infectious diseases is changeability, with change being a function of microbial, host, environmental, and societal changes that together translate into changes in the outcome of a host–microbe interaction. Given that species as varied as amphibians and bats can be threatened with extinction by microbes, the development of predictive tools for identifying microbial threats is both desirable and important.

## Virulence as an Emergent Property

To those familiar with the concept of emergence (Box 1), it probably comes as no surprise that microbial virulence is an emerging property. However, the traditional view of microbial pathogenesis has been reductionist [7], namely, assigning responsibility for virulence to either the microbe or the host. Such pathogen- and host-centric views, and in turn the scientific approaches fostered by these viewpoints, differ significantly in their historical underpinnings and philosophy [8]. In fact, neither alone can account for how new infectious diseases arise. The conclusion that virulence is an emergent property is obvious when one considers that microbial virulence can only be expressed in a susceptible host [9]. Consequently, the very same microbe can be virulent in one host but avirulent in another [10]. Furthermore, host immunity can negate virulence, as evidenced by the effectiveness of immunization that renders a microbe as deadly as the variola virus completely avirulent in individuals inoculated with the vaccinia virus. Infection with a microbe can result in diametrically opposed outcomes, ranging from the death of a host to elimination of the microbe. Hence, virulence is inherently novel, unpredictable, and irreducible to first principles.

**Box 1. The Concept of Emergent Properties**

Emergent properties are properties that cannot be entirely explained by their individual components [39]. An element of novelty is also considered to be an essential attribute of “emergent”, a term that contrasts with “resultant”, with the latter denoting an outcome that is predicted from the combination of the two components, such that resultant properties are additive whereas emergent properties are non-additive [20]. Another facet of emergent properties is that they are irreducible to their constituent components. Most treatises on emergence have emphasized that emergent properties have two components: an outcome that is greater than the sum of the parts and some form of novelty [20], [40], [41]. Although the concept of emergence dates back to antiquity when Aristotle stated that the “whole is not just the sum of its parts”, there is increasing interest in emergent properties as it becomes increasingly evident that reductionistic approaches cannot explain many phenomena in our world [42]. Examples of emergent properties in liquids are surface tension and viscosity, neither of which can be explained by analysis of individual molecules, as the properties pertain to the macroscopic world, and these phenomena have no corresponding analogs in the molecular realm. Biological systems have been described as characterized by emergent properties that exist at the edge of chaos, such that small fluctuations in their conditions can lead to sudden major changes [42]. Similarly, self-organized movements of individuals, as in schools of fish, can result in a variety of forms that are thought to protect against predators [43].

Critical to our understanding of virulence as a property that can only be expressed in a susceptible host is that both the microbe and the host bring their own emergent properties to their interaction. Host and microbial cells receive and process information by signaling cascades that manifest emergent properties [11]; e.g., gene expression studies reveal heterogeneous or bi-stable expression in clonal cell populations with important implications for phenotypic variability and fitness [12], [13]. Other emergent properties that have been identified in microbial and cellular systems could influence pathogenesis. Intracellular parasitism is associated with genome reduction, a phenomenon that could confer emergent properties, given that deliberate genome reduction in *E. coli* has led to unexpected emergent properties, such as ease of electroporation and increased stability of cloned DNA and plasmids [14].

On the host side, many aspects of the immune system have the potential to spawn emergent properties. The antigenic determinants of a microbe are defined by antibodies and processing by host cells, consequently existing only in the context of an immune system [15]. Microbial determinants can elicit host-damaging immune responses. Such deleterious responses exemplify a detrimental emergent property of the same host defense mechanisms that mediate antimicrobial effects. The outcome of a viral infection can depend on prior infection with related or unrelated viruses that express related antigens; hence, the infection history of a host affects the outcome of subsequent infections [16].

For those accustomed to viewing host–microbe interactions from an evolutionary perspective [17], the emergent nature of virulence is also no surprise, for the evolution of life itself can be viewed as an emergent process [18]. Even in relatively well-circumscribed systems such as Darwin's finches on the Galápagos Islands, evolutionary trends over time became increasingly unpredictable as a consequence of environmental fluctuations [19].

## Consequences of the Emergent Nature of Microbial Virulence

The fact that virulence is an emergent property of host, microbe, and their interaction has profound consequences for the field of microbial pathogenesis, for it implies that the outcome of host–microbe interaction is inherently unpredictable. Even with complete knowledge of microbes and hosts, the outcome of all possible interactions cannot be predicted for all microbes and all hosts. Lack of predictability should not be unduly discouraging. Even in systems in which emergent properties reveal novel functions, such as fluid surface tension and viscosity, recognition of these properties can be useful. For example, molecular structure might not predict the hydrodynamics of a fluid, but the empirical acquisition of information can be exploited to optimize pipeline diameter and flow rates. Novelty is unpredictable but novel events can be interpreted and comprehended once they have occurred [20]. A pessimist might argue that living systems are significantly more complex than flowing liquids. However, such pessimism may be unwarranted. The appearance of new influenza virus strains every year is an emergent property resulting from high rates of viral mutation and host selection of variants [21]. Hence, the time or place in which new pandemics will arise or the relative proportion of strains that will circulate each year cannot be predicted with certainty. Nevertheless, the likely appearance of new strains can be estimated from the history of population exposure to given strains and knowledge of recently circulating strains, and this information can be used to formulate the next year's vaccine.

## A Probabilistic Framework

Although the field of infectious diseases may never achieve the predictive certainty achieved in other branches of medicine, it may be possible to develop a probabilistic framework for the identification of microbial threats. Although all known pathogenic host–microbe interactions have unique aspects, and it is challenging to extrapolate from experiences with one microbe to another, a probabilistic framework can incorporate extant information and attempt to estimate risks. For example, the paucity of invasive fungal diseases in mammalian populations with intact immunity has been attributed to the combination of endothermy and adaptive immunity [22]. This notion could be extrapolated to other environmental microbes, i.e., those that cannot survive at mammalian temperatures have a low probability of emerging as new human pathogens. On the other hand, the identification of known virulence determinants in new bacterial strains may raise concern. In this regard, the expression of anthrax toxin components in *Bacillus cereus* produces an anthrax-like disease that is not caused by *Bacillus anthracis* [23].

Given the experience of recent decades, we can predict with confidence that new infectious diseases are likely to continue to emerge and make some general predictions about the nature of the microbes that could constitute these threats. One possibility is that an emergent pathogen could come from elsewhere in the animal kingdom. A comprehensive survey revealed that three-fourths of emerging pathogens are zoonotic [24]. Crossing the species barrier can result in particularly severe pathology, as pathogen and host have not had the opportunity to co-evolve toward equilibrium. Another good bet is that an RNA virus could emerge as a pathogen. The high mutation rate and generally broad host range of RNA viruses may favor species jumps [25], and many emergent human pathogens belong to this group, e.g., HIV, H5N1 influenza, SARS coronavirus, Nipah virus, and hemorrhagic fever viruses. On the other hand, global warming could hasten the emergence of new mammalian pathogenic fungi through thermal adaptation [26], given that the relative resistance of mammals to fungal diseases has been attributed to a combination of higher body temperatures and adaptive immunity [22], [27].

Despite abandoning hopes for certainty and determinism in predicting microbial pathogenic interactions, we can attempt to develop a probabilistic framework that endeavors to estimate the pathogenic potential of a microbe based on lessons from known host–microbe interactions. A variety of mathematical models based on game theory or quantitative genetics have been developed in attempts to understand the evolution of virulence [28], [29]. These have provided interesting new insights into host–pathogen interactions, including the tendency for evolutionary dynamics to produce oscillations and chaos rather than stable fitness-maximizing equilibria, the unpredictability that results when multiple games are played simultaneously, and the tendency for three-way co-evolution of virulence with host tolerance or resistance to select for greater virulence and variability [30]–[32].

## Preparing for the Unpredictable

Emerging infections seem to be becoming more frequent, and it is not difficult to understand why. An interesting experimental system examining a viral pathogen of moth larvae demonstrated that host dispersal promotes the evolution of greater virulence [33]. When hosts remain local, this encourages more “prudent” behavior by pathogens, but host movement encourages more infections and greater disease severity [34]. Global travel in the modern world can rapidly spread pathogenic microbes, but what is less obvious is that travel may also enhance virulence. Other factors contributing to the emergence and re-emergence of new pathogens include changes in land use, human migration, poverty, urbanization, antibiotics, modern agricultural practices, and other human behaviors [35], [36]. Microbial evolution and environmental change, anthropogenic or otherwise, will continue to drive this process. Another implication of the emergent nature of virulence is recognition of the hubris and futility of thinking that we can simply target resources to the human pathogens that we already know well. The discovery of HIV as the cause of AIDS [37] was greatly facilitated by research on avian and murine retroviruses that had taken place decades before [38], at a time when the significance of retroviruses as agents of human disease was unknown.

We share the view that sentinel capabilities are more important than predictive models at the present time [37], [38], but are optimistic that it will be possible to develop general analytical tools that can be applied to provide probabilistic assessments of threats from future unspecified agents. Comparative analysis of microbes with differing pathogenic potential and their hosts could provide insight into those interactions that are most likely to result in virulence. Hence, the best preparation for the unexpected and unpredictable nature of microbial threats will be the combination of enhanced surveillance with a broad exploration of the natural world to ascertain the range of microbial diversity from which new threats are likely to emerge.
